# Body mass-to-waist ratio strongly correlates with skeletal muscle volume in children

**DOI:** 10.1371/journal.pone.0177155

**Published:** 2017-05-05

**Authors:** Megumi Ohta, Taishi Midorikawa, Yuki Hikihara, Shizuo Sakamoto, Yasuo Kawakami, Tetsuo Fukunaga, Hiroaki Kanehisa

**Affiliations:** 1 School of International Liberal Studies, Chukyo University, Toyota, Aichi, Japan; 2 College of Health and Welfare, J.F. Oberlin University, Machida, Tokyo, Japan; 3 Faculty of Engineering, Chiba Institute of Technology, Narashino, Chiba, Japan; 4 Faculty of Sport Sciences, Waseda University, Tokorozawa, Saitama, Japan; 5 National Institute of Fitness and Sports in Kanoya, Kanoya, Kagoshima, Japan; University of Oslo, NORWAY

## Abstract

**Purpose:**

We hypothesized that body mass-to-waist ratio is strongly associated with the total-body skeletal muscle volume (SMV) in children. The purpose of the present study was to examine this hypothesis.

**Methods:**

By using magnetic resonance imaging, total-body SMV (SMV_MRI_) was determined in 70 boys and 53 girls aged 6 to 12 years. Waist was measured at each of the level of umbilicus (Wumb) and the minimum circumference (Wmin), and the ratio of body mass to each of the two measured values was calculated (BM/Wumb and BM/Wmin, respectively). A single regression analysis was used to examine the relationships between SMV_MRI_ and either BM/Wumb or BM/Wmin. On the basis of the obtained regression equations, SMV_MRI_ was estimated and referred to as SMV_BM/Wumb_ or SMV_BM/Wmin_.

**Results:**

In both boys and girls, SMV_MRI_ was highly correlated to BM/Wumb (r = 0.937 for boys and r = 0.939 for girls, *P* < 0.0001) and BM/Wmin (r = 0.915 and 0.942, *P* < 0.0001). R^2^ and the standard error of estimate for SMV_BM/Wumb_ were 0.878 and 706.2 cm^3^, respectively, in boys and 0.882 and 825.3 cm^3^, respectively, in girls, and those for SMV_BM/Wmin_ were 0.837 and 814.0 cm^3^, respectively, in boys and 0.888 and 804.1 cm^3^, respectively, in girls. In both boys and girls, there were no significant differences between SMV_MRI_ and either SMV_BM/Wumb_ or SMV_BM/Wmin,_ without systematic errors in Band-Altman plots. There was no significant effect of model on the absolute values of the residuals in both boys and girls.

**Conclusion:**

The current results indicate that body mass-to-waist ratio can be a convenient outcome measure for assessing the total-body skeletal muscle volume in children.

## Introduction

For children, accurate determination of total skeletal muscle mass within the whole body (total-body skeletal muscle mass) is essential for assessing nutritional status and basic factors involved in physical performances. As methods for quantifying the skeletal muscle mass or volume of the total-body, dual-energy x-ray absorptiometry (DXA) and magnetic resonance imaging (MRI) are extensively used in various research fields. These techniques are available for not only the adult population but also children and adolescents [1, 2, 3, 4, 5], but the instruments are expensive and are not portable. In addition, MRI determination of the total-body skeletal muscle mass is time consuming. Thus, measurements with DXA or MRI have limitations for application in field surveys examining large samples.

As an alternative approach, anthropometric prediction models have been developed (e.g., Lee et al. [6]). For children and adolescents, however, only two studies have addressed this subject [4, 5]. Poortmans et al. [4] developed a prediction equation for DXA-based total-body skeletal muscle mass using the product of height and the squared value of each of upper arm, thigh, and calf circumference corrected for subcutaneous adipose thickness (corrected circumference), sex (0 for female and 1 for male), and age as independent variables, being based on the corrected circumference model for predicting MRI-based total-body skeletal muscle mass in adults [6]. They reported that the developed anthropometric model was highly predictive (R^2^ = 0.97) for DXA-based total-body skeletal muscle mass. In addition, Qiterio et al. [5] validated and cross-validated two anthropometric models, being the corrected circumference model as described by Poortmans et al. [4] and a height-body mass model, for predicting DXA-based appendicular skeletal muscle mass in adolescent athletes. These findings support the utility of anthropometric variables for predicting total-body and appendicular skeletal muscle masses in children and adolescents.

Apart from the prediction of the total-body skeletal muscle mass, the validity of anthropometric variables for estimating body composition, notably fat mass and its value relative to body mass, have been extensively studied. Among anthropometric variables, waist circumference has been noted as a single outcome measure for assessing the total-body and abdominal adiposity in children and adolescents [7, 8, 9, 10, 11] as well as adults [12, 13]. Body mass is the sum of fat mass and fat-free mass. Fat-free mass is highly correlated to total-body skeletal muscle mass [14]. Taken together, it can be assumed that the ratio of body mass to waist circumference (BM/W) will become an index approximating the total-body skeletal muscle mass [15], because it can be translated to the following; BM/W 〜 (fat mass + fat-free mass) / (fat mass) 〜 1 + (total-body skeletal muscle mass) / (fat mass). Based on this idea, as BM/C becomes higher, the total-body skeletal muscle mass within body should be greater. WC as well as body mass is conveniently determined without special skills and apparatus for the measurements. If the aforementioned assumption is correct, BM/C can be a useful index for assessing the total-body skeletal muscle mass in population studies examining large samples. The present study aimed to elucidate this in children by using the total-body skeletal muscle volume (SMV) determined by MRI as reference data.

## Materials and methods

### Subjects

One hundred and twenty-three healthy boys (n = 70) and girls (n = 53) aged 6 to 12 yr voluntarily participated in this study. The physical characteristics of the subjects are summarized in Table 1. The average values of height and body mass for all subjects were similar to the normative data of Japanese boys and girls of the corresponding age group, reported by the Ministry of Education, Culture, Sports, Science and Technology. This study was approved by the ethics committee of the Faculty of Sport Sciences, Waseda University, and was consistent with their requirements for human experimentation. The subjects and their parents were fully informed about the procedures and the purpose of this study. Written informed consent was obtained from all participants and their parents.

**Table 1:** Physical characteristics of subjects.

Variables	Boys, n = 70			Girls, n = 53		
Age, yr	9.8	±	1.6	9.3	±	1.6
Height, cm	136.6	±	9.9	134.6	±	12.0
Body mass, kg	33.4	±	7.6	32.2	±	9.0
BMI, kg/m^2^	17.6	±	2.5	17.5	±	2.6
Wumb, cm	62.7	±	8.8	61.1	±	8.6
Wmin, cm	60.1	±	7.0	57.5	±	7.1
BM/Wumb, kg/m	52.8	±	6.6	52.1	±	9.1
BM/Wmin, kg/m	55.0	±	7.5	55.2	±	10.1
Total-body SMV, cm^3^	9042.6	±	2032.8	7962.3	±	2424.9

Values are presented as mean ±SD.

BMI, body mass index; Wumb, waist circumference at the umbilicus; Wmin, waist circumference at the minimum level; BM/Wumb, the ratio of body mass to Wumb; BM/Wmin, the ratio of body mass to Wmin; SMV, skeletal muscle volume.

### Anthropometric measurements

Height and body mass were measured in a standing position. Height was measured to the nearest 0.1 cm on a standard physician’s scale. Body mass was measured to the nearest 0.1 kg on a calibrated electric scale. Waist circumference was measured at each of the level of the umbilicus (Wumb) and the minimum circumference (Wmin) to the nearest 0.1 cm with a flexible metal tape (Flat rule, KDS, Japan). By using the two measures, BM/W was calculated (BM/Wumb and BM/Wmin, respectively).

### MRI measurements

A series of transverse images of the whole body was obtained by using MRI with a body coil (Signa 1.5T, GE Medical Systems, USA). In the MRI measurements, each subject lay supine with their legs extended and relaxed in the MRI device. Transverse scans were performed with a conventional T1-weighted spin-echo sequence, using multi slice sequences from the first cervical vertebrae to the malleolus lateralis (repetition time: 500 ms, echo time: 13.1 ms, slice thickness: 10 mm, interspaced distance: 0 mm, FOV: 48 cm, phase FOV: 0.6, matrix: 512 × 256). The number of excitations from the first cervical vertebrae to the femoral head was 1/2, and from the femoral head to the malleolus lateralis was 1. Five sets of acquisitions were obtained from the first cervical vertebrae to the femoral head while the subjects held their breath (approximately 20 s/set). The other three sets of acquisitions were obtained from the femoral head to the ankle joints during normal breathing. The total time required to acquire all of the MRI data for each subject was ~ 20 minutes, resulting in a total of approximately 100–150 slices for each subject. All images were traced by a highly trained technician, excluding the connective tissue, blood vessels, fat tissue and abdominal organs. MRI images were analyzed by ZedView software (LEXI Co. Ltd) for segmentation and calculation of cross-sectional tissue areas. Total-body SMV (cm^3^) was calculated by summing up the product of the calculated cross-sectional area (cm^2^) and the slice thickness (cm). The MRI-measured total-body SMV was referred to as SMV_MRI_. The coefficient of variation (%CV) for SMV_MRI_ measurements from a test–retest analysis was 2%.

### Statistical analysis

The normality of all measured variables was checked and subsequently confirmed by a Kolmogorov-Smirnov test. Descriptive values were presented as means ± SDs. A linear regression analysis was used to examine the relationships between SMV_MRI_ and either BM/Wumb or BM/Wmin. By using the obtained regression equations, total-body SMV was calculated and referred to as SMV_BM/Wumb_ or SMV_BM/Wmin_. The residual (SMV_MRI_−SMV_BM/Wumb_ or SMV_BM/Wmin_) of the estimate was plotted against the mean of SMV_MRI_ and each of SMV_BM/Wumb_ and SMV_BM/Wmin_ to confirm that no systematic error exist between the two variables, as described by Bland and Altman [16]. The standard error of estimate (SEE) was calculated to evaluate the accuracy of the estimate obtained by the developed prediction equation. The SEE was expressed as both an absolute value and relative to the mean value of SMV_MRI_. A linear regression analysis was conducted to examine the associations of the residual for each of SMV_BM/Wumb_ and SMV_BM/Wmin_ with age, height, body mass, body mass index (BMI). In addition to the aforementioned analyses, we tested the validity of the two BM/W models by using 10-fold cross-validation. In this analysis, fist, we randomized the order of the original data set for each of boys and girls, by using a random function from the spreadsheet software (Micrsoft Excell 2010, Microsoft CO., USA) [17], and then the randomized original data set was allocated into 10 subsets. The number of the subjects involved in a single subset was 7 for boys and 5 in 7 sub sets and 6 in 3 subsets for girls. One of 10 subsets was used as a testing set and the other 9 subsets were put together as a training set, and the holdout method was repeated 10 times. The average value of the residuals across all 10 trials was calculated. Furthermore, a stepwise multiple regression analysis, using SMV_MRI_ as the dependent variable and age, height, and body mass as independent variables, was performed to compare the accuracy of the estimate with that of the aforementioned single regressionanalysis. The estimated total-body SMV, based on the stepwise multiple regression analysis, was referred to as SMV_HBM_. For the SMV_HBM_, too, R^2^, SEE, and the residual were calculated and a Bland-Altman plot was applied to examine the existence of systematic error. In both single and multiple regression models, the residuals were also expressed as the absolute values and tested by a one-way repeated measurements analysis of variance (ANOVA) to confirm the effect of model on them. The significance level for statistical analysis was set at *P* < 0.05. All data were analyzed using a statistical software program (SPSS 21.0, IBM Co., Japan).

## Results

**Fig 1:**
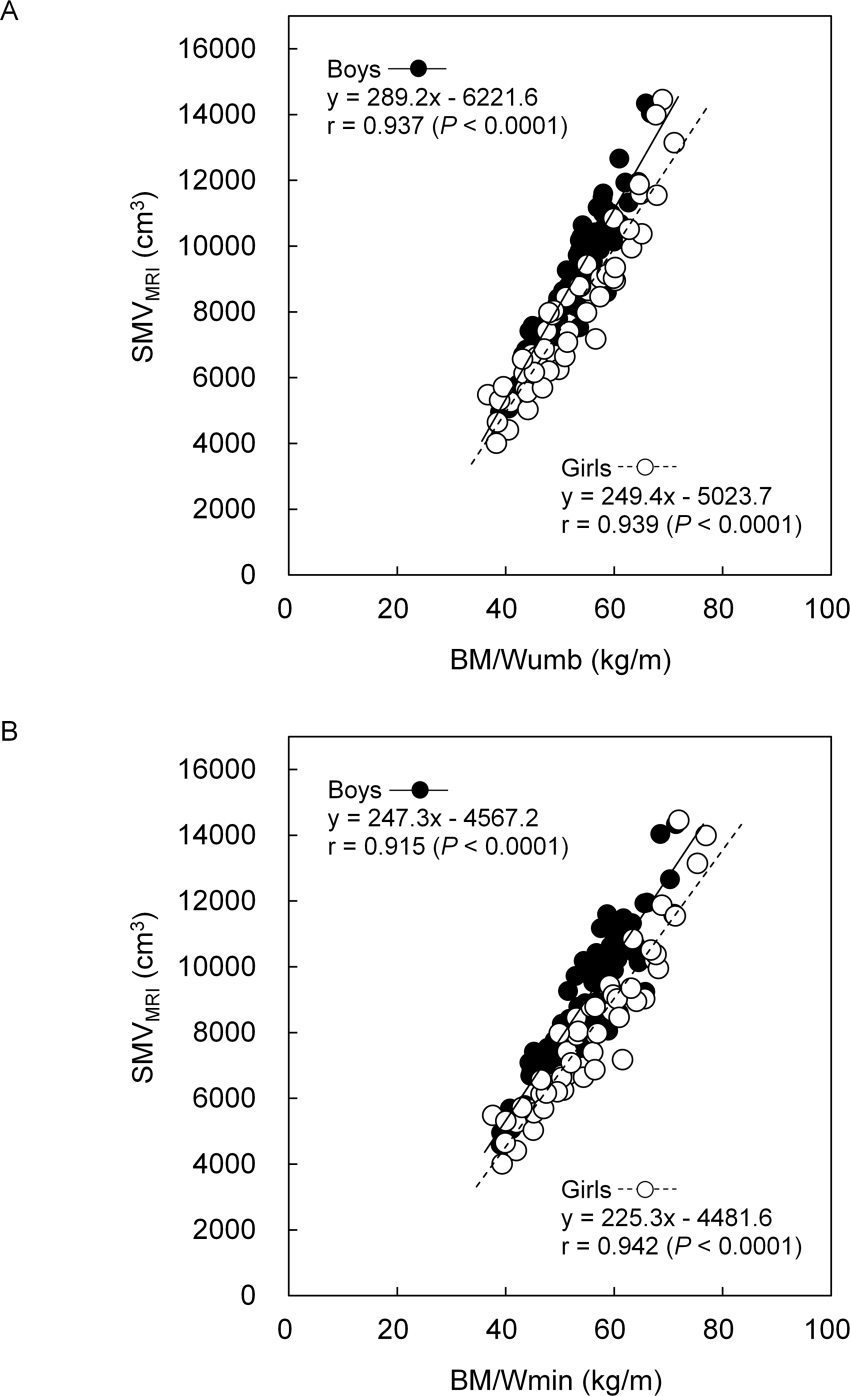
Relationships between SMV_MRI_ and both BM/Wumb (A) and Wmin (B).

Table 1 shows descriptive data for the measured variables. In both boys and girls, SMV_MRI_ was correlated to BM/Wumb (r = 0.937 for boys and r = 0.939 for girls, *P* < 0.0001) and BM/Wmin (r = 0.915 for boys and 0.942 for girls, *P* < 0.0001) (Fig 1). The SMV_MRI_ and SMV_BM/Wumb_ for boys were 9042.6 ± 1904.3 cm^3^ and 9043.2 ± 1860.0 cm^3^, respectively, and those for girls were 7962.3 ± 2277.1 cm^3^ and 7962.3 ± 2283.0 cm^3^, respectively. As a result of 10-fold cross-validation, the average residuals of SMV_MRI_ and SMV_BM/Wumb_ for boys were -0.2 cm^3^ and -3.0 cm^3^, respectively, and those for girls were -0.5 cm^3^ and 1.7 cm^3^, respectively.

**Fig 2:**
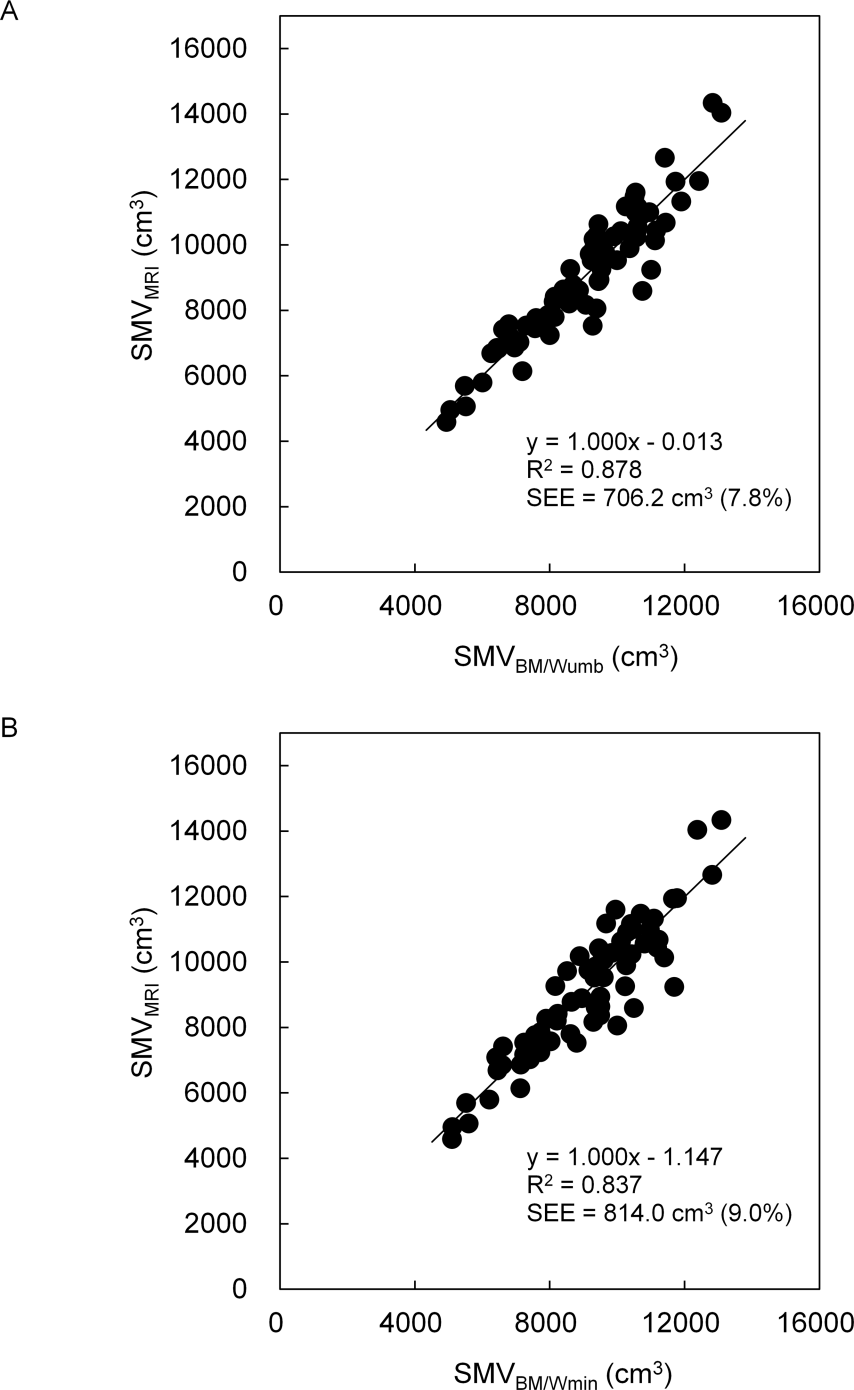
Relationships between SMV_MRI_ and both SMV_BM/Wumb_ (A) and SMV_BM/Wmin_ (B) in boys.

**Fig 3:**
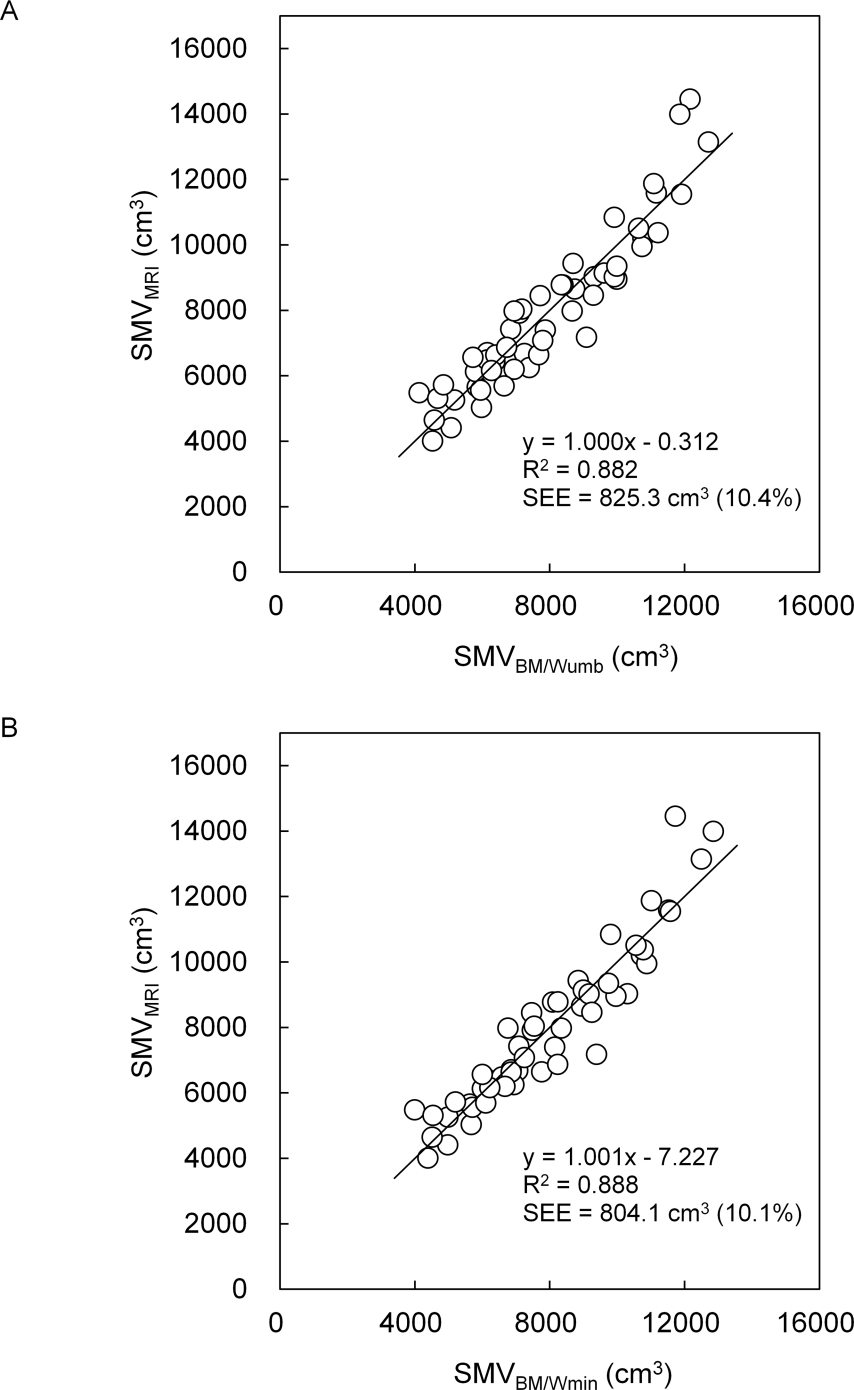
Relationships between SMV_MRI_ and both SMV_BM/Wumb_ (A) and SMV_BM/Wmin_ (B) in girls.

**Fig 4:**
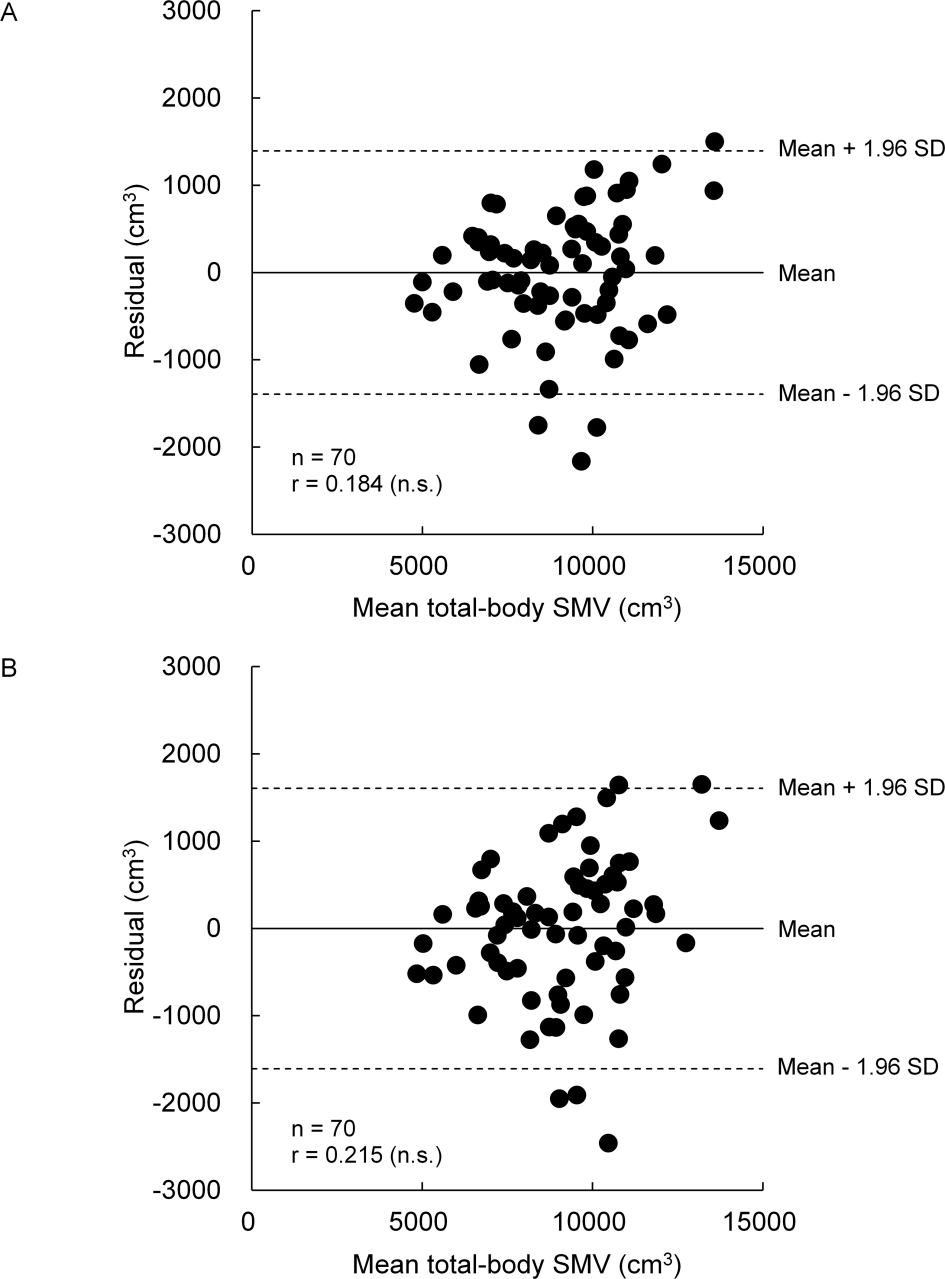
Relationships between residuals of each of SMV_BM/Wumb_ (A) and SMV_BM/Wmin_ (B) and the mean SMV determined by two methods in boys.

**Fig 5:**
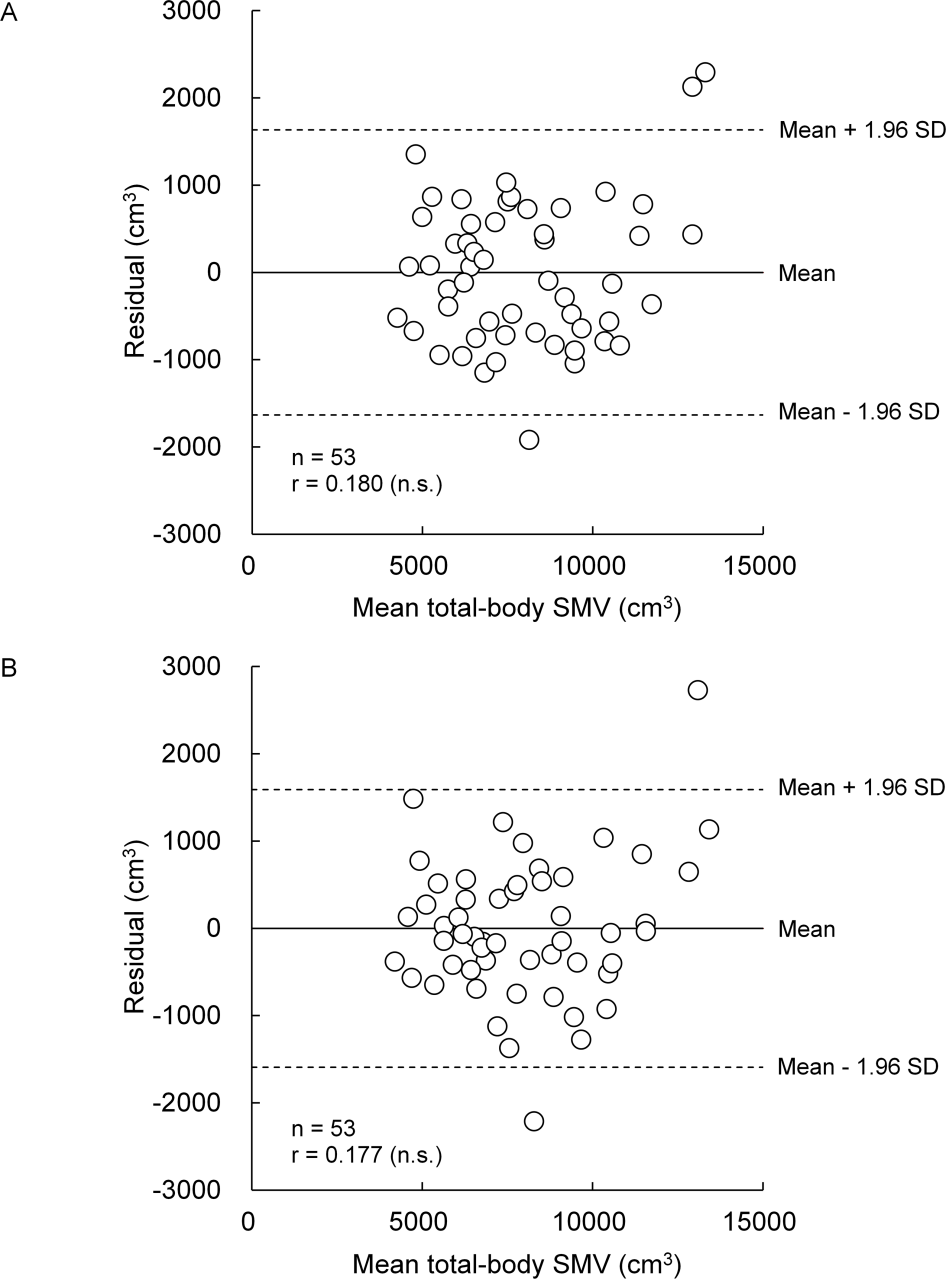
Relationships between residuals of each of SMV_BM/Wumb_ (A) and SMV_BM/Wmin_ (B) and the mean total-body SMV determined by two methods in girls.

Fig 2 and Fig 3 indicate the relationships between SMV_MRI_ and either SMV_BM/Wumb_ or SMV_BM/Wmin_ in boys and girls, respectively. R^2^ and SEE for SMV_BM/Wumb_ were 0.878 and 706.2 cm^3^ (7.8%), respectively, in boys and 0.882 and 825.3 cm^3^ (10.4%), respectively, in girls, and those for SMV_BM/Wmin_ were 0.837 and 814.0 cm^3^ (9.0%), respectively, in boys and 0.888 and 804.1 cm^3^ (10.1%), respectively, in girls. In both indexes and sexes, no significant systematic errors were found in Bland-Altman plots (Figs 4 and 5).

In boys, the residuals of SMV_BM/Wumb_ had no significant associations with age, height, body mass, and body mass index (BMI), but those of SMV_BM/Wmin_ were significantly associated with age (r = 0.287, *P* < 0.05) and BMI (r = -0.314, *P* < 0.01). In girls, the residuals of SMV_BM/Wumb_ showed a significant association with BMI (r = 0. 296, *P* < 0.05), but those of SMV_BM/Wmin_ were not significantly correlated with age and the anthropometric variables.

Multiple regression analysis produced prediction equations for boys and girls as follows:

$$\text{boys}:\;SMV_{MRI}\left(cm^{3}\right)=\left(age\times 205.9\right)+\left(height\times 119.6\right)+\left(body\;mass\times 75.1\right)–11808.3$$

$$\text{girls}:\;SMV_{MRI}\left(cm^{3}\right)=\left(height\times 95.7\right)+\left(body\;mass\times 139.4\right)–9404.7$$

where age, height, and body mass are in year, centimeter, and kilogram, respectively. The R^2^ and SEE of these equations were 0.882 and 692.9 cm^3^ (7.7%), respectively, in boys and 0.910 and 721.6 cm^3^ (9.1%), respectively, in girls. The SMV_HBM_ was 9042.6 ± 1909.2 cm^3^ in boys and 7962.3 ± 2313.0 cm^3^ in girls. There were no significant systematic errors in Bland-Altman plots {r = 0.180 (*P* = 0.137) for boys and r = 0.156 (*P* = 0.267) for girls}. There was no significant effect of model on the absolute values of the residuals in both boys (*F* = 2.105, *P* = 0.126) and girls (*F* = 2.180, *P* = 0.118).

## Discussion

The present study newly proposed body mass-to-waist ratio as an outcome measure for conveniently assessing total-body SMV in children. As expected, BM/Wumb and BM/Wmin were strongly correlated to SMV_MRI_ in both boys and girls. The average values of SMV_BM/Wumb_ and SMV_BM/Wmin_ were almost the same as that of SMV_MRI_, and, as a result of 10-fold cross-validation, the average values of residuals were so small as they can be ignored (-3.0–1.7 cm^3^). In addition, Bland-Altman plots showed that the residuals of SMV_BM/Wumb_ and SMV_BM/Wmin_ were independent of the magnitude of SMV. These results indicate that the body mass-to-waist ratio presented here can be an outcome measure for assessing the total-body SMV in children of both sexes.

The present study is the first case examining the applicability of anthropometric measurements for estimating the total-body SMV in children by using MRI-based SMV as the reference data. As described earlier, two studies have examined the validity of anthropometry-based prediction for total-body [4] or appendicular [5] skeletal muscle mass in children and adolescents. In their results, the prediction equations for total-body and appendicular skeletal muscle masses in children and adolescents were highly validated with satisfactory confidence. For their findings, it should be noted that the two studies used DXA-based lean tissue mass as a reference variable representing skeletal muscle mass. Certainly, appendicular lean tissue mass can be strongly related to the total-body skeletal muscle mass measured by MRI in children and adolescents (R^2^ = 0.98, SEE = 0.57 kg) [1]. However, it has also been shown that appendicular lean tissue mass for children and adolescents involves a higher portion of non-muscle lean soft tissue such as skin, connective tissue, and the lean portion of adipose tissue, compared with that for adults [1]. This implies that a prediction equation with DXA-based lean tissue mass as a dependent variable will involve a potential error for estimating total-body skeletal muscle mass. At the same time, the difference in the reference data (i.e., MRI-based SMV vs. DXA-based lean tissue mass) makes it difficult to directly compare the accuracy of the estimate in the current study with that in the previous studies cited above.

To the best of our knowledge, available information on the applicability of the anthropometric model for predicting the MRI-based total-body skeletal muscle mass is limited to the report of Lee et al. [6] who used an adult population. They developed prediction equations based on the corrected circumference and height-body mass models with SEE values of 2.2 kg and 2.8 kg, respectively. Based on their data, the calculated relative value of SEE was 8.2% in the corrected circumference model and 10.5% in the height-body mass model. In the comparison based on the relative value of SEE, the accuracy of the BM/W model presented here is comparable to that in the previous study. Apart from anthropometric model, Midorikawa et al. [3] and Kim et al. [1] have developed ultrasound-based muscle thickness and DXA-based appendicular lean tissue models, respectively, on the basis of MRI-based skeletal muscle volume or mass. The SEE values observed here are similar to those in the ultrasound-based muscle thickness model for boys (659 cm^3^, 7%) and girls (731 cm^3^, 9.5%) [3]. However, it is also true that the accuracy of the BM/W model is lower than that in the prediction equation for MRI-based total-body skeletal muscle mass, using DXA-based appendicular lean tissue mass as an independent variable (SEE = 0.565 kg, the calculated relative value of SEE = 5%) [1].

For estimating total-body skeletal muscle mass, the corrected circumference model needs to measure the circumference and subcutaneous adipose thickness of each of the upper arm, thigh, and calf. Considering the convenience of anthropometric measurements, Lee et al. [6] and Qiterio et al. [5] developed the height-body mass model. In their results, the accuracy of height-body mass model for predicting total body [6] and appendicular [5] skeletal muscle mass was similar to that of the corrected circumference. In the present study, the anthropometric variables needed to develop the corrected circumference model were not measured. However, we examined whether the accuracy of BM/W model for estimating total-body SMV differs from that of height-body mass model. In the current results, the observed SEE values were almost the same between the two models. Furthermore, there were no significant differences in the absolute values of the residuals among the models. Taking these results into account together with the previous findings cited above, the accuracy of estimation of the BM/W model may be assumed to be similar to those in not only height-body mass but also the corrected circumference models. The measurements of waist circumference as well as height and body mass can be easily performed without expensive apparatus and specific skill training for data sampling. In the sense of test burden, it will be equal between BM/W and height-body mass models. As described earlier, however, waist circumference is a single measure for approximating adiposity [7, 8, 9, 10, 11]. As compared to height-body mass model, therefore, the measurement of waist circumference and the calculation of BM/W will be of practical means to assess conveniently both total-body SMV and adiposity.

In the current results, there was no significant difference between SMV_BM/Wumb_ and SMV_BM/Wmin_ in the absolute value of the error of the estimate. This indicates that the accuracy of the estimate is almost the same between the two BM/W models. However, the associations of the residuals of SMV_BM/Wumb_ and SMV_BM/Wmin_ with BMI differed between boys and girls. Namely, BMI was negatively correlated with the residuals of SMV_BM/Wmin_ for boys and positively correlated with the residuals of SMV_BM/Wumb_ for girls. This implies that, for individuals with greater BMI, SMV_BM/Wmin_ for boys was overestimated and SMV_BM/Wumb_ for girls was underestimated. As similar as waist circumference, BMI is also extensively used as an outcome measure for approximating the total-body adiposity in population studies. Thus, the current result indicates that the accuracy of SMV_BM/Wmin_ for boys and SMV_BM/Wumb_ for girls is affected by the magnitude of the total-body adiposity. In the present study, we assumed that waist circumference and BM/W would reflect total-body adiposity and muscularity, respectively. For this idea, the associations observed between the residuals and BMI provide a problem that, in both boys and girls, there might be a difference between Wumb and Wmin in the magnitude of their associations with total-body adiposity. However, this is canceled out by previous findings indicating that, in children [18] and adults [19] of both sexes, the magnitude of the association between waist circumference and the total-body fat mass is independent of the site where waist circumference is determined, although the absolute values of the waist circumference differ between the sites. In any case, the current results suggest that, as a practical issue, the use of Wumb and Wmin for boys and girls, respectively, enables assessment of the total-body SMV without influence of BMI.

In previous studies examining the validity of anthropometry-based prediction equations for total skeletal muscle mass [6, 4] or appendicular lean soft tissue mass [5], age was selected as a significant predictor. In the current study, age was significantly associated with SMV_MRI_ in both boys (r = 0.747, *P* < 0.0001) and girls (r = 0.681, *P* < 0.0001). Thus, there is a possibility that adding age to BM/W would improve the accuracy of estimation for SMV. To examine this, we applied a stepwise multiple regression analysis to develop a equation for estimating SMV_MRI_ with age and BM/Wumb or BM/Wmin as independent variables. As a result, for the girls, age was not selected as a significant variable. In contrast, for the boys, multiple regression analysis produced prediction equations with age and either SMV_BM/Wumb_ (R^2^ = 0.886, SEE = 686.8 cm^3^) or SMV_BM/Wmin_ (R^2^ = 0.862, SEE = 752.8 cm^3^) as independent variables. In the two equations, the observed R^2^ and SEE were similar to those in the equations with only BM/W. In addition, the absolute values of the residuals in the multi regression models were not significantly different from those in the equations with the BM/W model. In the current results, age was significantly correlated with the two BM/W values in both boys and girls (r = 0.690–0.779, *P* < 0.0001). In addition, the age range for the subjects examined here was from 6 to 12 years. It has been shown that the cross-sectional areas (CSAs) of limb muscles increase up to about 17 years of age in both boys and girls [20]. The growth curves of muscle CSAs were similar between both sexes up to 13 years, but boys had greater increments than girls at and after 13 years [20]. Thus, it is likely that the ages of the subjects examined in the present study would be prior to those in which boys show a greater increase in muscle mass. Taking these aspects into account, it seems that, at least for the subjects examined here, adding age to BM/W might not have contributed to improve the accuracy of estimating SMV_MRI_ beyond the level of the BM/W model.

The present study used only Japanese boys and girls as the subjects. Thus, whether the current findings can be applied to other age and/or ethnic groups remains question. Form the report of Abe et al. [15], BM/W was significantly correlated to the total-body skeletal muscle mass, estimated from ultrasound-based muscle thickness measurements, in men and women aged 60–80 years. This partially supports to assume that BM/WC can be an index approximating the total-body skeletal muscle mass in the elderly. Even so, however, there is a need to clarify the accuracy of the estimate for the corresponding age groups. In addition, it is known that there are ethnic differences in waist circumference and adiposity in children and adolescents [21] as well as adults [22]. Camhi et al. [22] observed race and sex differences in the relationships of WC to visceral, subcutaneous, and total-body fat. Furtheremore, the present studies determined WCs at two different sites. As described earlier, the magnitude of waist circumference varies with its measurement site [17, 18]. These aspects suggest that regression equation in the relationship between BM/W and total-body skeletal muscle volume would differ with race examined and measurement site adopted for waist circumference. Further studies are needed to elucidate these issues.

In conclusion, the current results indicate that the body mass-to-waist ratio can be an index for assessing total-body skeletal muscle volume in children. Further study with a cross-validation sample, including different age and ethnic groups, is essential to generalize the current findings.
